# Performance of diagnostic tests for *Trypanosoma brucei brucei* in experimentally infected pigs

**DOI:** 10.1371/journal.pntd.0011730

**Published:** 2023-11-09

**Authors:** Kadidiata Ilboudo, Alain Boulangé, Robert Eustache Hounyèmè, Geoffrey Gimonneau, Jacques Kaboré, Adrien Gaston Marie Belem, Marc Desquesnes, Veerle Lejon, Mathurin Koffi, Vincent Jamonneau, Sophie Thévenon

**Affiliations:** 1 Unité de Recherche sur les Maladies à Vecteurs et Biodiversité, Centre International de Recherche-Développement sur l’Elevage en zone Subhumide, Bobo-Dioulasso, Burkina Faso; 2 Unité de Formation et de Recherche en Sciences de la Vie et de la Terre, Université Nazi Boni, Bobo-Dioulasso, Burkina Faso; 3 Unité de Recherche « Trypanosomoses », Institut Pierre Richet, Bouaké, Côte d’Ivoire; 4 CIRAD, UMR INTERTRYP, Montpellier, France; 5 INTERTRYP, University of Montpellier, CIRAD, IRD, Montpellier, France; 6 Laboratoire National d’Élevage et de Recherches Vétérinaires, Service de Bio-Écologie et Pathologies Parasitaires, Dakar—Hann, Sénégal; 7 Institut du Développement Rural, Université Nazi Boni, Bobo-Dioulasso, Burkina Faso; 8 National Veterinary School of Toulouse (ENVT), Toulouse, France; 9 Laboratoire de Biodiversité et Gestion des Ecosystèmes Tropicaux, Unité de Recherche en Génétique et Épidémiologie Moléculaire, UFR Environnement, Université Jean Lorougnon Guédé, Daloa, Côte d’Ivoire; Universidade Federal de Minas Gerais, BRAZIL

## Abstract

Animal African trypanosomosis is an important vector-borne disease of livestock in sub-Saharan Africa. Pigs seem relatively tolerant to trypanosome infection and could act as a reservoir of trypanosomes affecting animals and humans. Our ability to reliably detect trypanosome infection in pigs depends on the performance of diagnostic tools, which is not well known. In pigs experimentally infected with *Trypanosoma brucei brucei*, we evaluated the performance of parasitological Buffy Coat Technique (BCT), two molecular (TBR and 5.8S PCR) and four serological tests (CATT, HAT Sero-*K*-Set rapid diagnostic test–RDT, indirect ELISA, immune trypanolysis). Most diagnostic tests showed high specificity, estimated at 100% (95% CI = 74–100%) with the exception of CATT and RDT whose specificity varied between 100% (95% CI = 74–100%) to 50% (95% CI = 7–93%) during the experiment. The sensitivity of each test fluctuated over the course of the infection. The percentage of positive BCT over the infection (30%) was lower than of positive PCR (56% and 62%, depending on primers). Among the serological tests, the percentage of positive tests was 97%, 96%, 86% and 84% for RDT, ELISA, immune trypanolysis and CATT, respectively. Fair agreement was observed between both molecular tests (κ = 0.36). Among the serological tests, the agreement between the ELISA and the RDT was substantial (κ = 0.65). Our results on the *T*.*b*. *brucei* infection model suggest that serological techniques are efficient in detecting the chronic phase of infection, PCR is able to detect positive samples several months after parasites inoculation while BCT becomes negative. BCT examination and RDT are useful to get a quick information in the field, and BCT can be used for treatment decision. ELISA appears most suited for epidemiological studies. The selection of diagnostic tests for trypanosomosis in pigs depends on the context, the objectives and the available resources.

## Author summary

The presence of wild and domestic healthy carriers is an obstacle to the control and elimination of animal and human trypanosomosis in Africa. Domestic pigs are receptive to infection by most trypanosomes, and may act as a reservoir of parasites for livestock and humans. Indeed, field studies highlighted that pigs are often positive for different diagnostic tests for trypanosome infections. However, the performances of these tests are poorly known in pigs, while accurate diagnosis of pig trypanosomosis is essential for epidemiological surveillance and implementation of control measures. This study evaluated the diagnostic performance of one parasitological, two molecular and four serological diagnostic tests to detect *Trypanosoma brucei brucei* infection in experimentally infected pigs. The results showed a high specificity of most tests, except for the serological tests CATT and HAT Sero-*K*-Set Rapid Diagnostic Test (RDT). The sensitivity varied highly depending on the tests.

While the choice of a test or a test combination depends on the objectives of a given study and the available resources, parasitological observation can be proposed for quick field screening in conjunction with a RDT, provided that RDT performances are confirmed in a larger study, and indirect antibody-ELISA and PCR are suited for epidemiological investigations.

## Introduction

Animal African trypanosomosis (AAT), or nagana, is considered as one of the main health constraints to livestock production in sub-Sahara Africa [1], with an additional indirect impact on crop production [2]. This disease is caused by unicellular protozoan parasites of the genus *Trypanosoma* (order Kinetoplastida). *Trypanosoma brucei brucei*, *T*. *congolense* and *T*. *vivax* are the main species responsible for AAT in livestock [3,4]. They are mainly transmitted cyclically by tsetse flies (*Glossina* spp.) [5], but *T*. *vivax* can also be transmitted mechanically by biting flies such as *Tabanus* spp and *Stomoxys* spp [6,7].

For epidemiological studies, surveillance, planning and implementation of control measures, diagnosis of AAT is based on direct or indirect trypanosomes detection tests [8], since clinical manifestations of AAT can be confused with other parasitic affections or signs of malnutrition, vary by strain and species of the parasite, and also depend on host factors [9]. Direct trypanosome detection by microscopy is commonly considered as the gold standard for confirmation of infection as it is highly specific, though the technique can lack sensitivity [10]. Serological tests based on the detection of specific antibodies, and molecular tests that detect parasite DNA have shown high sensitivities and specificities [10]. The evaluation of these diagnostic tests has so far mainly been carried out on cattle [11,12] that are susceptible to the infection and for which the economic impact of AAT is important [1,2].

Yet, in order to achieve a sustainable control of AAT, it is crucial to consider that different wild and domestic host species may act as reservoir of parasites. The case of local African pigs is particularly challenging since pigs are (i) particularly receptive and generally tolerant to most of the African trypanosomes infections [13–19] and (ii) the preferred host for tsetse, especially *Glossina palpalis palpalis* [20,21]. In the field, pigs have been found positive for *T*. *brucei* in different locations in Africa [14,16–18,22–24]. *Trypanosoma brucei brucei* is considered less pathogenic than *T*. *congolense* and *T*. *vivax* in cattle and small ruminants, and *T*.*b*. *brucei* infections are often asymptomatic in pigs [3]. However, attention should also be paid to *T*.*b*. *brucei*. First, *T*.*b*. *brucei* can be responsible for anaemia and severe disease in cattle [25,26], small ruminants [27] and pigs [28,29] leading to death [30]. Second, because it has the ability to leave the blood circulation and invade deep tissues [31,32], *T*.*b*. *brucei* can be difficult to detect by direct blood observation and might escape from trypanocidal drug action [33]. In addition, its differential diagnosis with *T*.*b*. *rhodesiense* and *T*.*b*. *gambiense*, responsible for Human African Trypanosomiasis (HAT) [34] is difficult even though pigs may carry *T*.*b*. *rhodesiense* [22] and are suspected to be a reservoir of *T*.*b*. *gambiense* [35]. In the interest to be able to detect *T*. *brucei* s.l., and to distinguish afterwards the different parasite subspecies, the performances of the diagnostic tests must be accurately evaluated. In the field, parasitological observations, PCR and serological tests have been used in pigs to assess both the circulation of animal trypanosomes and of human-infecting trypanosomes, but without a previous knowledge of their performances in terms of sensitivity and specificity [18,36].

In this context, this study aims at evaluating the diagnostic performance of diagnostic tools developed for AAT and HAT to detect *T*.*b*. *brucei* infection in pigs. For this purpose, we carried out an experimental infection of pigs with *T*.*b*. *brucei* and assessed the performances of seven diagnostic tests: parasitological examination based on the Buffy Coat Technique (BCT), two molecular PCR tests and four serological tests: indirect ELISA, immune trypanolysis (TL), Card Agglutination Test for Trypanosomiasis*/T*.*b*. *gambiense* (CATT)—and HAT Sero-*K*-Set rapid diagnostic test (RDT). The experimental infection as well as the BCT and TL results have already been described in a publication focusing on the *T*.*b*. *gambiense* specificity of TL using LiTat 1.3 and LiTat 1.5 variant antigen types (VAT) [37]. This study suggested that TL using the LiTat 1.6 VAT had sufficient sensitivity to be used as a serological test for the diagnosis of *T*.*b*. *brucei* infections in pigs [37]. The CATT and HAT Sero-*K*-Set are field tests for HAT diagnosis, but since comparable field tests do not exist for *T*.*b*. *brucei*, they were included in the evaluation assuming potential cross-reactivity. In the present study, the performance of the tests was evaluated in terms of sensitivity and specificity. Also taking into account test feasibility, we can conclude that the selection of diagnostic tests for *T*.*b*. *brucei* in pigs depends on the context, the objectives and the available resources.

## Material and methods

### Ethics statement

The study protocol was approved by the *Comité d’Ethique et de Biosécurité* of CIRDES (Centre International de Recherche-Développement sur l’Elevage en zone Subhumide) and the implementing authorisation (070_2020/ADM/DG/nka) was obtained in June 2020. Infected pigs were kept in mosquito net protected stables to avoid contact with potential vectors of trypanosomes. Pigs were observed daily by a veterinarian and any animal showing signs of distress or suffering (weight loss) during the experimental period was euthanized. All animals were euthanized at the end of the experiment.

### Experimental infection and sampling

The 6 months experimental infection was already described elsewhere [37]. Briefly, 12 pigs were acquired from a tsetse-free area in western Burkina Faso. Eight were infected with the *T*.*b*. *brucei* MSUS/CI/2013/BE8P2P2 strain and four constituted the uninfected control group.

Peripheral blood samples for BCT were collected for each animal from the auricular vein in a 70 μl heparinised capillary tube (75 x 1.5 mm) the day of infection (D0), 7 days post infection (DPI) and twice a week from 7 to 189 DPI. Blood samples were also collected in 5 ml EDTA vacutainer tubes from the central vascular system, 19 days before infection (-19 DPI), 5 days before infection (-5 DPI), the day of infection (D0) and then weekly until 189 DPI. After performing the CATT and the RDT, the remaining blood was centrifuged at 15.000 rpm for 15 min. One ml plasma and 500 μl buffy coat were collected and stored at -20°C for subsequent serological (indirect ELISA, TL) and molecular (PCR) analyses, respectively.

### Diagnostic tests

For parasitological examination, the capillary tube was examined by the BCT [38]. For molecular diagnosis, DNA was processed from the buffy coat samples using the Chelex 100 DNA preparation method [39]. Briefly, 500 μl of buffy coat was mixed with 500 μl of 5% Chelex 100 (Bio-Rad, Foster City, CA, USA) in a 1.5 ml Eppendorf tube. The tube was then incubated at 56°C for 1 h, then at 95°C for 30 min, and centrifuged at 12 000 x g for 3 min, and the supernatant containing DNA was used as a template for PCR. PCR amplification was performed using TBR1/2 (5’-CGAATGAATATTAAACAATGCGCAG-3’/ 5’-AGAACCATTTATTAGCTTTGTTGC-3’) [40], and 5.8SF/R primers specific of *Trypanosomatidae* (5’-GCGATGGATGACTTGGCTTC-3’/ 5’-TCCCATGCGCCGTTTGCGTTC-3’). These recently designed -in house- 5.8S primers, amplify a short fragment (114bp) of the 5.8S ribosomal DNA and were evaluated here for the first time taking advantage of the experimental conditions. The PCR reactions were carried out in a final volume of 25 μl, composed of molecular biology grade water (16,9 μl), 10X buffer containing 25 mM MgCl2 (2.5 mM final, 2.5 μl), dNTP (deoxyribonucleotide triphosphate) (10 mM, 1 μl), primer (10 mM, 1 μl for each), 0.1 μl of 5 U/μl of FIREPol *Taq* DNA polymerase (0.5 unit final) and 2.5 μl of template to be tested. The reactions were performed with an initial step of 15 min at 95°c, followed by 35 cycles of 94°C (30 s), 55°C (30 s), and 72°C (30 s) for TBR PCR, and 35 cycles of 94°C (1 min), 50°C (1 min), and 72°C (2 min) for 5.8S PCR, both finishing by an extension at 72°C (5 min). Amplified products were visualized with UV light after electrophoresis in a 2% agarose gel.

Four serological tests were performed. The indirect-ELISA (ELISA) protocol for the detection of antibodies against *Trypanosoma* was adapted from the World Organisation for Animal Health (founded as OIE) protocol and standardised for the analysis of pig plasma [41,42]. *Trypanosoma brucei brucei* Farakoba 80/CRTA/1 strain [43] soluble antigens (whole trypanosomes lysate, WTL,) [44] diluted in 50 mM carbonate buffer pH 9.6 at a concentration of 5 μg/ml were used to coat 96-well Polysorp NUNC plates. 100 μl of this dilution was placed in each well and kept overnight at 4°C. The next day, after blocking of the plate for 30 min at 37°C, plasma samples were first diluted 1/20, and then diluted 1/10 during their transfer to the wells with 10 μl of pre-diluted plasma in 90 μl of blocking buffer (PBS with 0.1% Tween 20 and 5% skimmed milk) and each sample was deposited in duplicate, as were positive (strongly and weakly) and negative reference samples. After an incubation time of 1 h at 37°C, the plates were emptied and washed three times with washing buffer (PBS with 0.1% Tween 20). Peroxidase conjugate (anti-pig IgG HRPO BIO-RAD) was diluted to 1/20000 in blocking buffer and was added to the well (100 μl /well) and incubated for 30 minutes. After incubation the plates were emptied, washed 3 times and 100 μl of ABTS (L-tartaric acid/sodium carbonate) was added to each well. After 30 min at 37°C in the dark, the plates were read at 405 nm wavelength using an ELISA reader. The relative percentage of positivity (RPP) of each sample was calculated from the optical density of the sample in relation to optical densities of positive and negative standards [45]. Any sample with a RPP less than or equal to 20% was considered negative, while a sample with a RPP higher than 20% was considered positive.

The immune trypanolysis (TL) was performed to test plasma samples as previously described [46]. In the present study, we will only take into account the results obtained using cloned populations of *T*.*b*. *gambiense* VAT LiTat 1.6 (TL LiTat 1.6), as that VAT showed the highest sensitivity for the detection of *T*.*b*. *brucei* in pigs [37]. Briefly, 25 μl of plasma was mixed with an equal volume of guinea pig serum, to which 50 μl of a 10^7^ trypanosomes/ml suspension prepared from infected mouse blood was added. After 90 min of incubation at room temperature, the suspension was examined by microscopy (×250). Trypanolysis was considered positive when 50–100% of the trypanosomes were lysed.

For the CATT [47], twenty-five μl of EDTA blood was mixed with a drop of CATT reagent on the agglutination card, and spread over the reaction zone. The mixture was then placed on a rotary agitator for 5 min at 60 rounds per minute. The reaction was considered positive in presence of blue agglutinations [47,48].

The HAT Sero-*K*-Set RDT (Coris BioConcept, Belgium) was used following the manufacturer instructions. Twenty-five μl of fresh EDTA blood was dispensed in the sample well and two drops of buffer were added. The result was read 15 minutes later.

### Statistical analysis and evaluated parameters

Data were entered into Excel and analysed with the R software version 4.0.4 (2021-02-15). The ggplot2 function [49] was used to generate graphs. The specificity and sensitivity of the different tests, and their confidence interval (CI), were assessed with the binomial test. Specificity, (the ability of a test to give a negative result in pathogens-free animals), was determined on the 12 pigs before infection and from the four non-infected ones after infection. Sensitivity, (the ability of a test to give a positive result in infected animals), was assessed over time on the eight infected pigs from 7 DPI. The Friedman test, a non-parametric test for repeated data or block design, and Wilcoxon test for paired data were used to assess the significance of the difference in the date of apparition of the first positive results [50]. Comparison of the sensitivity of the tests was evaluated during the infection using a Generalized Additive Model (gam model) with the R packages mgcv [51,52]. The gam model allows to consider the repeated data at the animal level and to estimate a non-linear temporal effect with smoothing parameters. Parasitological and molecular tests (BCT and both 5.8S and TBR PCR) were compared using the PCR 5.8S as the reference test from 7 DPI, and serological tests (RDT, CATT, ELISA, TL LiTat 1.6) were compared using RDT as the reference test after 7 DPI. Test result (positive or negative) was modelled according to a binomial function (logit link), the diagnostic test effects on the intercept and smoothing factor were assessed, and pig effect was adjusted as random smooths. In addition, agreements between diagnostic tests were assessed by kappa statistic (κ) which quantifies the agreement between two tests for the analysed samples beyond chance and ranges from 0 (no agreement beyond chance) to 1 (perfect agreement beyond chance) using the R package irr [53–55]. Kappa statistics were calculated in the infected group from 7 DPI.

The files containing the raw data and the R script can be found in the S1 Text.

## Results

### Individual response of the pigs to the parasitological diagnostic test

**Fig 1:**
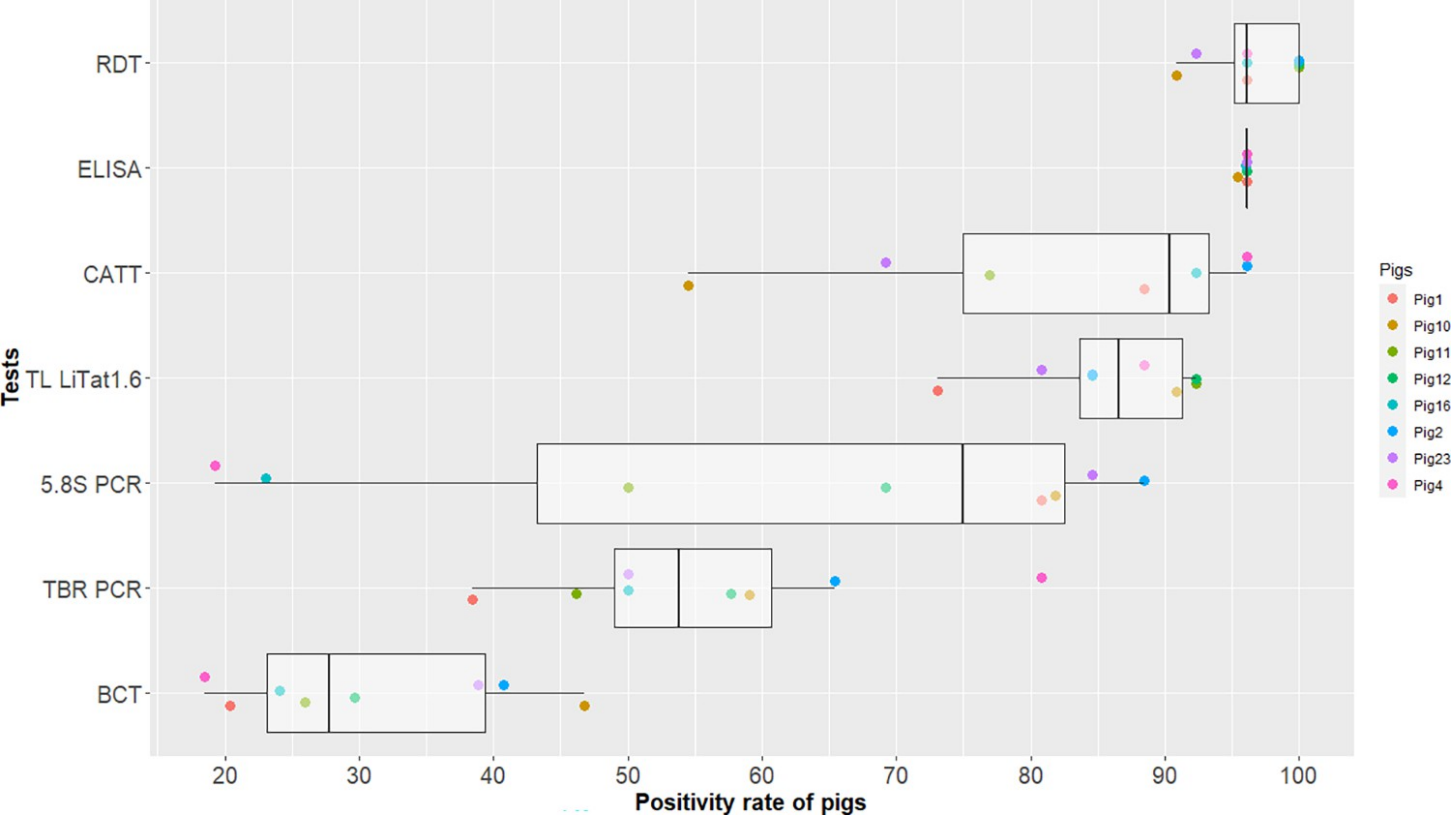
Boxplot of the percentages of positive time points to each test estimated per pig. The points represent the percentages of positive tests for each pig of the infected group estimated from 7 DPI until the end of the experiment. The central line represents the median, the box the interquartile range. Each pig is represented by a specific color.

BCT results have been previously published [37]. Briefly, a total of 642 samples of which 426 came from the infected group (8 pigs) and 216 from the control group (4 pigs) were tested. In the control group, no trypanosomes were detected during the experiment. All pigs in the infected group were trypanosome positive during the experiment. The first positive tests were observed between 10 and 14 DPI (average 11±2 DPI, median at 10 DPI). The percentage of positive time points in BCT from DPI 7 until the end of the experiment per pig ranged from 19% (95% CI = 9–31% for pig 4) to 47% (95% CI = 31–61% for pig 10) (Fig 1).

### Individual result of the pigs in the molecular diagnostic tests

**Fig 2:**
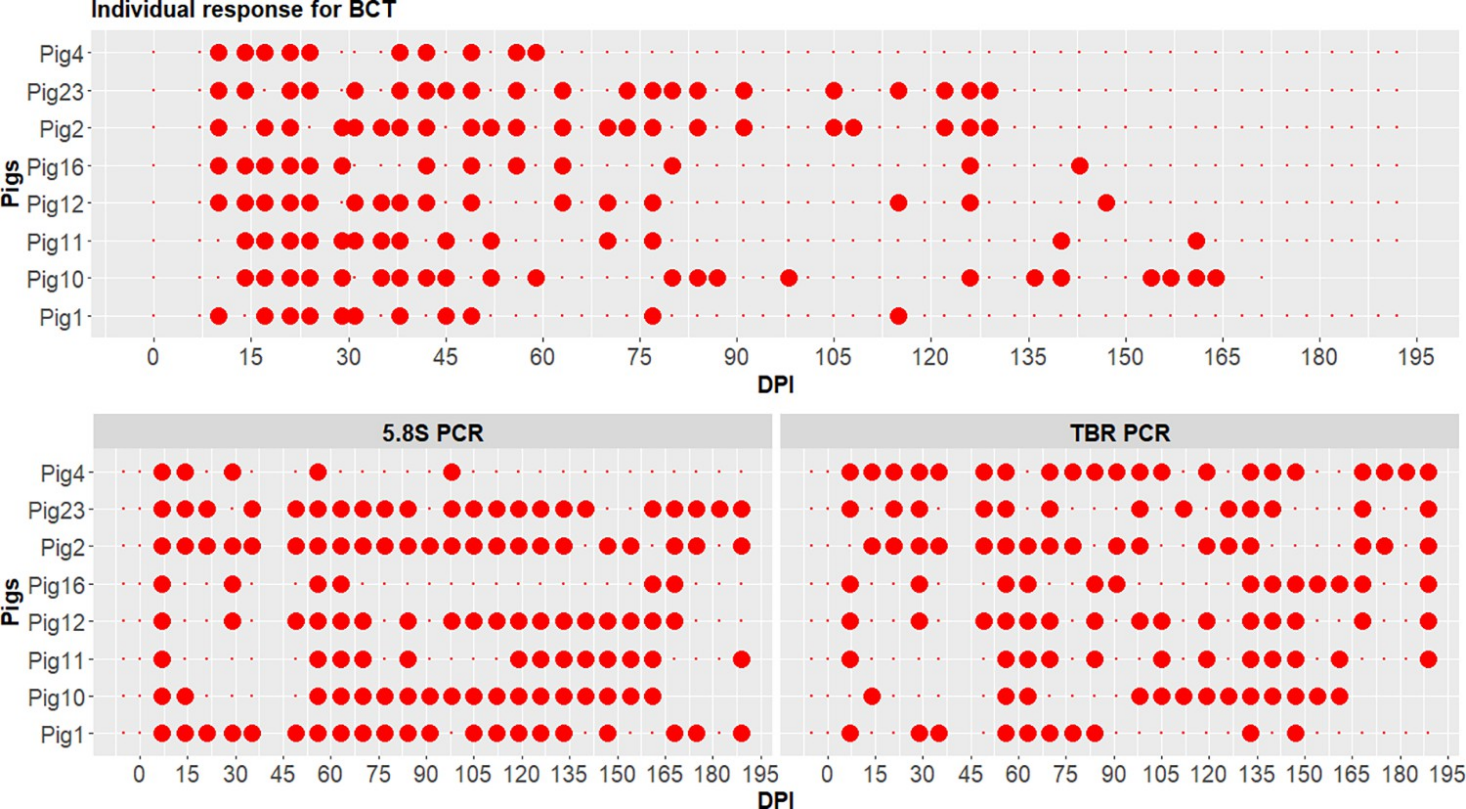
Individual result of the eight pigs of the infected group in BCT and PCR tests. Top: positivity in BCT, Bottom left: positivity in 5.8S PCR. Bottom right: positivity in TBR PCR. Large red points: positive test; small red points: negative test; DPI: days post infection.

Molecular tests were not performed on the -19 DPI samples and Pig 10 was euthanized at 175 DPI. Both the TBR and the 5.8S PCR were performed on a total of 332 samples of which 220 samples were from the infected group and 112 from the control group. No positive PCR was observed in the control group. Fig 2 shows the individual results of each pig of the infected group in the molecular tests. All infected pigs showed positive results for both PCRs. Infection was detected early with 5.8S PCR, all pigs being positive at 7 DPI. With TBR PCR, the first positive tests were detected between 7 and 14 DPI, with a median first date of positivity of 7 DPI and an average of 9±3 DPI. However, during the course of infection, there was a large variability in the percentage of positive time points between pigs, the percentage ranging from 38% (95% CI = 20–59% for pig 1) to 81% (95% CI = 61–93% for pig 4) for TBR, and from 19% (95% CI = 7–39%, for pig 4) to 88% (95% CI = 70–98% for pig 2) for 5.8S PCR (Fig 1). Positive tests were observed until the end of the experiment at 189 DPI, with TBR PCR (pigs 2, 4, 11, 12, 16, 23) and 5.8S PCR (pigs 1, 2, 11, 23).

### Individual results of the pigs in the serological tests

**Fig 3:**
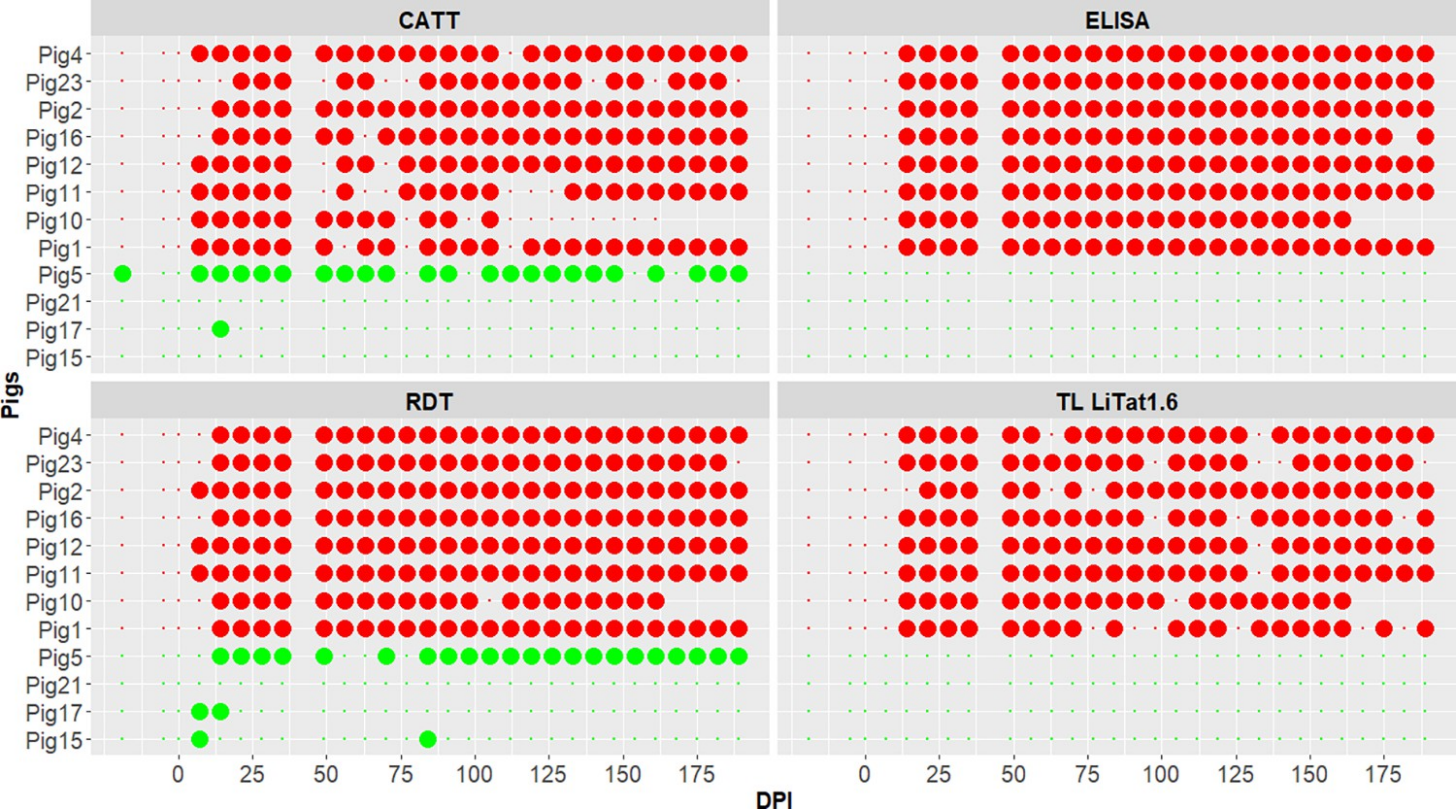
Individual result of all pigs to the four serological tests (ELISA, TL LiTat 1.6, RDT and CATT). Large points: positive test; small points: negative test. Pigs of the infected group are indicated in red, pigs of the control group in green. DPI: days post infection.

The number of analysed samples of the uninfected group was 116 for all tests. For the infected group, there were 225 and 227 plasma samples tested by ELISA and TL LiTat 1.6, respectively, and 228 blood samples tested by CATT and RDT (Pig 10 was euthanized at 175 DPI). The difference in the number of tests was due to an insufficient quantity of collected plasma. The individual result of pigs in the four serological tests is shown in Fig 3. TL LiTat 1.6 results have been previously published [37]. No positive tests were found by ELISA or TL LiTat 1.6 in the control group. However, pig 5 of the control pigs was tested positive in CATT and RDT on a regular basis during the course of the experiment. Pig 17 was positive once with CATT and twice with RDT, while pig 15 was positive twice with RDT only. Only pig 21 remained negative in both CATT and RDT.

All infected animals gave positive results in every test at various time points during infection (Fig 3). The first positivity occurred from 7 to 14 DPI for RDT (average 11±4 DPI, median 14), from 7 to 21 DPI for CATT (average 11±5 DPI, median 7), from 14 to 21 DPI for TL LiTat 1.6 (average 15±2 DPI, median 14), and at 14 DPI for ELISA for all animals.

The percentage of positive time points in ELISA per pig, from 7 DPI, was 96% (95% CI = 80–100) (Fig 1). Individual results observed in RDT were also homogenous, with a percentage of positive time points varying from 91% (95% CI = 71–99% for pig 10) to 100% (95% CI = 87–100% for pigs 2, 11, 12). The percentages of positive time points were more variable between pigs for TL LiTat 1.6 and CATT, from 73% (95% CI = 52–88% for pig 1) to 92% (95% CI = 75–99% for pigs 11, 12) and from 55% (95% CI = 32–76% for pig 10) to 96% (95% CI = 80–100% for pig 2, 4) respectively.

### Overall diagnostic performance of the different tests

Dates of first detection of positivity differed significantly between tests (P-value<10^−3^ for the Friedman test), but P-values of paired tests could not be exactly computed due to many ties and zeros. We could just notice a trend of earlier appearance of positivity with molecular tests (PCR 5.8S and TBR) and CATT and RDT, followed by BCT and finally ELISA and TL LiTat 1.6 (Figs 2 and 3).

As no positive samples were detected with BCT, both TBR and 5.8S PCR, ELISA and TL LiTat 1.6 in non-infected pigs, the specificities of these tests were estimated at 100% (95% CI = 74–100%). CATT and RDT displayed positive results in non-infected pigs hence their specificities varied from 100% (95% CI = 74–100%, estimated on 12 pigs before infection) to 50% (95% CI = 7–93%, estimated in the control group) according to the sampling day.

**Fig 4:**
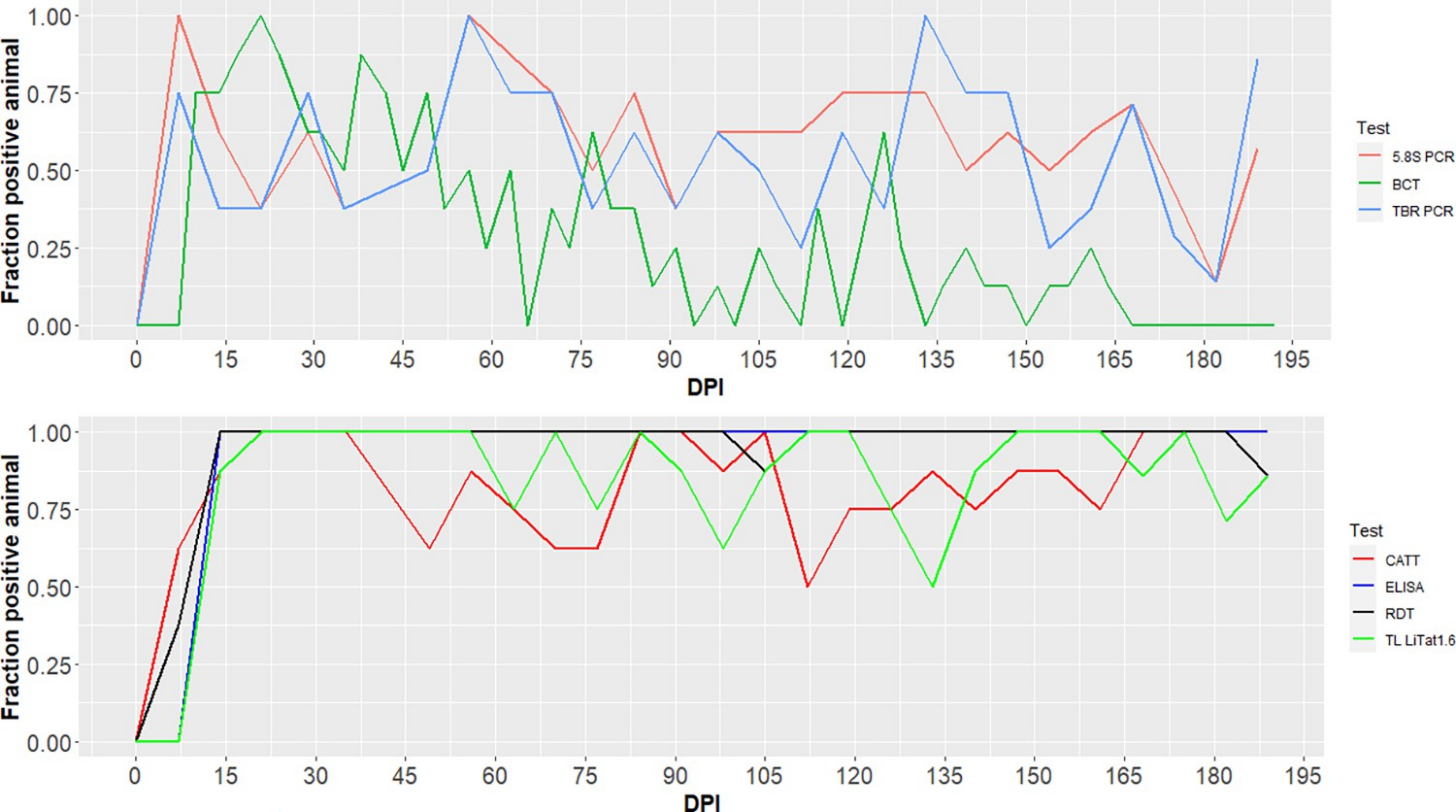
Evolution of the estimated sensitivity during the experiment for each diagnostic test (up parasitological and PCR tests, down serological tests). DPI: days post-infection. For a minimal sensitivity of 0%, 95% Confidence Interval (CI) = 0–37%, for a maximal sensitivity of 100%, 95% CI = 63–100%, for a medium sensitivity of 50%, 95% CI = 16–84%.

Sensitivities differed greatly between parasitological, molecular and serological techniques. An estimation of the sensitivity of each test throughout infection is illustrated in Fig 4.

The Table 1 presents the specificity and the sensitivity of the diagnostic tests, as well as the percentage of negative and positive tests averaged over the experiment, calculated on non-infected and infected animals respectively. Sensitivity of the BCT varied a lot over the course of the infection, ranging from 0% (95% CI = 0–37%) at 7 DPI and at the end of the experiment, to 100% (95% CI = 63–100%), the percentage of positive tests being 30% over the whole experiment (calculated from 7 DPI). The sensitivity of molecular tests also fluctuated a lot in the course of infection but remained above 0%, and the percentage of positive tests was 56% and 62% for TBR and 5.8S respectively over the whole experiment. After first antibodies detection at 14 DPI, sensitivities remained high and stable for ELISA and RDT, with 96% and 97% of positive tests respectively. The sensitivities of CATT and TL LiTat 1.6 were 50% (95% CI = 16–84%) to 100% (95% CI = 63–100%) from 14 DPI, the percentage of positive tests being 84% and 86% for CATT and TL LiTat 1.6 respectively from 7 DPI.

**Table 1:** Specificity and sensitivity of different diagnostic tests. Specificity and the percentage of negative tests were estimated during the experiment in non-infected animals (all animals before infection and in the control group), the first one was assessed overtime and the last one averaged on all the samples. Sensitivity and the percentage of positive tests were estimated during the experiment in the infected group from 7 DPI, the first one was assessed overtime and the last one averaged on all the samples. Regarding specificity and sensitivity, minimal and maximal values observed during the experiment are shown on the first and second lines respectively, if they are different. 95% confidence intervals are indicated in brackets.

Tests	Specificity	Percentage of negative tests	Sensitivity	Percentage of positive tests
BCT	100% (74–100%)	100% (98–100%)	0% (0–37%) 100% (63–100%)	30% (26–35%)
TBR PCR	100% (74–100%)	100% (97–100%)	14% (0–58%) 100% (63–100%)	56% (43–63%)
5.8S PCR	100% (74–100%)	100% (97–100%)	14% (0–58%) 100% (63–100%)	62% (55–68%)
RDT	50% (7–93%) 100% (74–100%)	81% (74–87%)	0% (0–37%) 100% (63–100%)	97% (93–98%)
ELISA	100% (74–100%)	100% (97–100%)	0% (0–37%) 100% (63–100%)	96% (92–98%)
CATT	50% (7–93%) 100% (74–100%)	83% (76–87%)	50% (16–84%) 100% (63–00%)	84% (78–89%)
TL LiTat1.6	100% (74–100%)	100% (97–100%)	0% (0–37%) 100% (63–100%)	86% (80–90%)

Sensitivity was first compared for the parasitological and molecular tests using the gam model. TBR PCR did not significantly differ in term of sensitivity from the 5.8S PCR (P-value = 0.13 on the intercept and P-value = 0.80 on the smoothing terms using the gam model). On the contrary, the BCT sensitivity was significantly lower than the one of 5.8S PCR (estimate = -1.98, P-value = 10^−14^ on the intercept and P-value = 10^−15^ on the smoothing terms).

For serological tests, ELISA did not significantly differ in sensitivity from RDT (P-value = 0.99 on the intercept and on the smoothing terms using the gam model), while CATT and TL LiTat 1.6 showed a significant lower sensitivity than RDT (effect = -2.87, P-value = 10^−4^ on the intercept for CATT, and effect = -2.45, P-value = 10^−3^ on the intercept for TL LiTat 1.6).

### Concordance between the different diagnostic tests

The data on the agreement between diagnostic tests are presented in Table 2. Agreement was estimated on the one hand for BCT and PCR tests. It was poor between the two PCR tests and the BCT (0<κ<0.20). Fair agreement was observed between the two PCR tests (κ = 0.36). On the other hand, among the serological tests, the agreement was poor between ELISA and CATT, CATT and TL LiTat 1.6, CATT and RDT (0<κ<0.20). Conversely, there was moderate agreement between the ELISA and the TL LiTat 1.6 (κ = 0.41) and substantial agreement between the ELISA and the RDT (κ = 0.65).

**Table 2:** Agreement between molecular and parasitological tests on the one hand, and between serological diagnostic tests on the other hand, estimated in the infected group. Kappa range interpretation: <0 = Disagreement, 0–0.2 = poor agreement, 0.21–0.4 = fair agreement, 0.41–0.6 = moderate agreement, 0.61–0.8 = substantial agreement, 0.81–1 = almost perfect agreement.

Pair of compared tests	Kappa (κ)	Interpretation
TBR PCR-BCT	0.004	Poor
5.8S PCR-BCT	0.10	Poor
TBR PCR-5.8S PCR	0.36	Fair
ELISA-RDT	0.65	Substantial
ELISA-CATT	0.08	Poor
ELISA-TL LiTat 1.6	0.41	Moderate
CATT-TL LiTat 1.6	0.05	Poor
CATT-RDT	0.10	Poor
RDT-TL LiTat 1.6	0.35	Fair
ELISA-TBR PCR	-0.04	Disagreement
CATT-TBR PCR	-0.10	Disagreement
RDT-TBR PCR	-0.05	Disagreement
TL LiTat 1.6-TBR PCR	-0.04	Disagreement
ELISA-5.8S PCR	-0.08	Disagreement
CATT-5.8S PCR	-0.23	Disagreement
RDT-5.8S PCR	-0.07	Disagreement
TL LiTat 1.6–5.8S PCR	-0.10	Disagreement

## Discussion

The diagnosis of trypanosomoses is a basic requirement for epidemiological studies as well as for the implementation and follow-up of control measures. Compared to cattle, pigs are often an overlooked species since they usually express marginal symptoms of trypanosomosis [13,56]. However, paying attention to pigs is essential for AAT control and sustainable elimination of HAT, as pigs may act as a potential reservoir of both animal and human trypanosomes [35]. The prevalence of trypanosomes in pigs living in tsetse-infested areas should therefore be monitored as accurately as possible, which requires efficient diagnostic tools. Our study is the first to evaluate the diagnostic performance of seven tests developed for AAT or HAT including one parasitological, two molecular and four serological tests, in pigs experimentally infected with *T*.*b*. *brucei*.

All tests showed high specificity, reaching up to 100% (95% CI = 74–100%), with the exception of CATT and RDT whose specificity was assessed between 50% (95% CI = 7–93%) to 100% (95% CI = 74–100%) due to some positive samples in non-infected pigs, especially in one pig which was positive to CATT and RDT for most of the experiment while remaining negative to BCT, both PCR, ELISA and TL (including other VATs LiTat 1.3 and LiTat 1.5 [37]). These false positives are likely attributed to cross-reactions with antibodies targeting other pathogens [57]. The SD Bioline HAT RDT [58], another serological test initially developed for HAT diagnosis, also showed a low specificity when used in animals [59].

Furthermore, CATT and HAT Sero-*K*-Set RDT developed for HAT diagnosis are based on variant antigens of *T*.*b*. *gambiense* [60,61]. In humans, their specificity ranges from 88% to 99% depending on the study [47,48,60,62,63]. The use of diluted plasma instead of whole blood could improve specificity, at the expense of sensitivity, as it is proposed for humans [64]. We chose to use whole blood in the present study, implementing the CATT as a simple field test for antibody detection.

We also compared the dates at which tests became positive for the first time, which defines the prepatent period. The infection was detected the earliest by molecular tests at a median of 7 DPI for 5.8S PCR and TBR PCR, respectively, as compared to parasitological detection by BCT occurring between 10 and 14 DPI (median at 10 DPI). Early detection of infection by PCR were reported in experimental studies using different trypanosomes and hosts species [12,65,66]. Specific antibodies were first detected by CATT (7–21 DPI) and RDT (7–14 DPI), followed by ELISA (14 DPI) and TL LiTat 1.6 (14–21 DPI). The delay of detection in serology was expected according to the delay of production of specific antibodies and based on results obtained in experimental infection with *T*. *vivax* and *T*. *evansi* [12,67].

The sensitivity of the tests was also assessed. First, the BCT, which is amongst the most sensitive parasitological tests [68,69], was compared to molecular tests (TBR and 5.8S PCR). The percentage of positive tests with BCT (30%) was lower than with TBR PCR (56%) and 5.8S PCR (61%), and BCT was significantly less sensitive than molecular tests. PCR has been reported to be more sensitive than parasitological techniques in experimental conditions [12,65,66,70,71] and when applied to field samples [16,72,73]. We also observed that the BCT sensitivity decreased from 30 DPI onward. BCT false negative results are common when parasitaemia is low in peripheral blood, below the detection threshold [11], and it has a low sensitivity in the field [24,74]. Sensitivities of PCRs were also highly fluctuating, though always higher than BCT. Interestingly, while all pigs were negative to BCT during the last month of the experiment, molecular tests kept giving positive results, highlighting the persistence of the trypanosomal DNA in the bloodstream. A higher sensitivity of the TBR PCR versus 5.8S PCR was expected, on the basis of previous studies that compared amplification of the satellite DNA, thought to be present in 10,000 to 20,000 copies per genome [75–77], and ribosomal DNA [70,78], repeated around 100–200 times per genome [77,79]. Difference in primers affinity for the targeted sequences could explain this observation. Alternatively, diversity in the sequence targeted by TBR primers could explain a lack of sensitivity of the TBR PCR, as suggested by Van Reet et al. (2021) [80]. Performances of the new 5.8S PCR will deserve complementary analyses to accurately assess its analytical sensitivity and specificity in different types of samples, coming from various host species and infection types.

When comparing sensitivity of antibody detection tests, it appeared that all serological tests successfully detected *T*.*b*. *brucei* infection in pigs. The responses were more stable for ELISA and RDT with 96% and 97% of positive tests respectively, when calculated from 7 DPI. However, from 14 DPI, the sensitivity of ELISA was 100% until the end of the experiment. Overall, CATT and TL LiTat 1.6 showed lower sensitivities than did RDT and ELISA, and their sensitivity also fluctuated during the course of the experiment. The ELISA antibody-detection test developed here used whole soluble antigens of *T*.*b*. *brucei*. Such a test making use of WTL has generally high sensitivity [12] as it provides numerous antigen targets that can be recognized by antibodies. The fluctuating sensitivity of the CATT during the chronic phase could be due to the fact that CATT detects mainly IgM. For example, in cattle trypanosomosis due to *T*. *congolense*, IgM rates were lower than IgG rates. As previously shown [37], sensitivity of TL LiTat 1.6 for *T*.*b*. *brucei* infection in pigs was high, but it seemed less than for surveillance of gambiense-HAT [46,81], for which TL was developed. Overall, higher sensitivity of serological tests than of BCT and molecular tests was expected and can be explained by the sustained production and persistence of specific antibodies, whereas the parasitaemia is fluctuating [82,83].

Agreements among the methods varied from poor (no agreement between positive and negative results of a pair of tests for a same sample) to substantial (agreement between positive and negative results). Agreement between molecular tests and BCT was poor. That some samples were positive to one or two molecular tests and negative to BCT could be expected from the difference in their sensitivities [11]. However, some parasitology-positive samples were negative by PCR. Parasitological examinations were done on peripheral blood collected from the auricular vein, while PCR was performed on blood collected from the anterior vena cava. Local concentration of parasites could differ depending on blood origin explaining this result. More surprising was the fair agreement between both PCR. These unexpected results may be due to technical failures related to the DNA preparation method or amplification inhibiting factors [84]. ELISA and RDT showed a high agreement while CATT provided low agreement with other serological tests, maybe suggesting fluctuating IgM concentrations. As expected, Kappa indexes calculated between serological and molecular tests were negative, highlighting disagreement between the two types tests that identify totally different elements, host antibodies and parasite DNA respectively that present different dynamics, but that can thus be viewed as complementary.

Given the relatively small number of animals, as well as the individual variations between them, the observed indicators of performance had large confidence intervals and should be interpreted with caution. The use of experimental infections may somehow bias the estimated diagnostic sensitivity and specificity compared to natural infections [85]. Indeed, a better specificity for all tests can be expected for experimental infections, as pigs were initially treated against various infections, while in the field animals remain exposed to many pathogens, including other trypanosome species. Sensitivity could be also biased in experimental infection due to the use of a specific trypanosome strain, the inoculation dose and the administration route, that differ from natural infections. Furthermore, it must be noted that, to precisely assess the prepatent period, samples should have been collected more frequently during the first three weeks of the experiment, which could not be performed due to the stress caused to pigs.

Despite these limitations, we can provide some conclusions on tests performance and recommendations to the potential use of these diagnostic tests to assess pig infection by *T*.*b*. *brucei*. High specificities were recorded for BCT, both PCRs, ELISA and TL LiTat1.6, but observed specificities were lower for RDT and CATT. On the other hand, ELISA and TDR showed high sensitivities, followed by TL LiTat1.6 and CATT. The PCRs displayed a moderate sensitivity, and BCT a poor one, especially in late phase of infection. Serological techniques are efficient in detecting infected animals, especially during the chronic phase of infection, but it must be reminded that a positive test does not necessarily reflects active infections since antibodies may persist after treatment or self-cure [86,87]. PCRs were able to detect positive samples until six months post-inoculation while parasitaemia had become undetectable, signalling the persistent presence of trypanosomes. The joint use of both PCRs (TBR and 5.8S), run in parallel, strengthened the global sensitivity of our molecular tests, the cumulated percentage of positive tests reaching 75%, thus decreasing the global risk of getting false-negative results without deteriorating the specificity. In terms of practical use, BCT, TDR and CATT can be performed in the field. TDR is a so-called point of care test, CATT is suitable to quickly screen dozens of animals, while BCT requires only basic equipment, a centrifuge and a microscope, and is easy to perform by a trained staff. ELISA, PCR and TL require a well-equipped laboratory and well-trained technicians. In addition, TL needs animal facilities to multiply live parasites and must therefore be performed in a reference laboratory [88].

In conclusion, the selection of tests to be used to detect *T*.*b*. *brucei* in pigs will depend on the context, the objectives and the financial and technical resources available according to the elements mentioned above. Parasitological detection by BCT and the use of RDT and/or CATT can be promoted in the field to identify early infections and support treatment decision for BCT, and to have an immediate impression of the epidemiological situation for CATT and RDT. ELISA appears to be the most reliable and suitable laboratory serological test for epidemiological surveys and for the monitoring of control campaigns during which prevalence is expected to decrease if control is efficient. The use of PCR is also important to identify ongoing infections and is adequate to assess the presence of animal reservoirs of trypanosomes. For that purpose, the use of two pairs of primers targeting different DNA sequences could be proposed. It should be kept in mind that performances of the tests for other trypanosome species infecting pigs have not been assessed here and remain to be compared. Furthermore, as our result suggest that CATT and RDT lack specificity, additional research is needed to develop point-of-care diagnostics that should be accurate, cheap, and easy to use [89].

## Supporting information

S1 Table diagnostic_BCT.csv. Raw data for buffy coat test. Columns header: Index (index line), Date: date of sampling; Pig: pig identifier; Group: group C for the control group, I for infected group; Result1: result of the BCT test 0 for negative, 1 for positive; DPI: Date Post-Infection; Phase: pre_inf for before infection; inf for from infection; Status: healthy or infected status of pigs; PA.ml: log10 of parasitemia in trypanosomes/ml.
(CSV)

S2 Table diagnostic_PCR.csv. Raw data for PCR tests. Columns header: Index (index line), Date: date of sampling; Group: group C for the control group, I for infected group; Pig: pig identifier; Test: molecular test (TBR PCR, 5.8S PCR); Result: result of the PCR test 0 for negative, 1 for positive; DPI: Date Post-Infection; Phase: pre_inf for before infection; inf for from infection; Status: healthy or infected status of pigs.
(CSV)

S3 Table diagnostic_Serologic.csv. Raw data for serological tests. Columns header: Index (index line), Date: date of sampling; Group: group C for the control group, I for infected group; Pig: pig identifier; Test: serolgoical test (CATT, RDT, ELISA, TL LiTat1.6); Result: result of the serological test 0 for negative, 1 for positive; DPI: Date Post-Infection; Phase: pre_inf for before infection; inf for from infection; Status: healthy or infected status of pigs.
(CSV)

S4 Table Prepatente_all_diag.csv. Table containing first date of positivity for each pig and each diagnostic tests. Pig: pig identifier; Test: diagnostic tests (BCT, TBR PCR, 5.8S PCR, CATT, RDT,ELISA, TL LiTat1.6); PP: time point of first positivity in DPI; Group: C for the control group, I for infected group.
(CSV)

S1 Text Script_diagnostic_tools.R. R script for statistical analyses.
(R) Click here for additional data file.
